# A Needle in A Haystack: Tracing Bivalve-Associated Viruses in High-Throughput Transcriptomic Data

**DOI:** 10.3390/v11030205

**Published:** 2019-03-01

**Authors:** Umberto Rosani, Maxwell Shapiro, Paola Venier, Bassem Allam

**Affiliations:** 1 Department of Biology, University of Padua, 35121 Padua, Italy; umberto.rosani@unipd.it (U.R.); paola.venier@unipd.it (P.V.); 2 Department of Applied Mathematics and Statistics, Stony Brook University, Stony Brook, NY 11794-5000, USA; maxwell.shapiro@stonybrook.edu; 3 School of Marine and Atmospheric Sciences, Stony Brook University, Stony Brook, NY 11794-5000, USA

**Keywords:** bivalve, virome, RNA-seq, RNA viruses, sncRNA, ADAR, RNAi

## Abstract

Bivalve mollusks thrive in environments rich in microorganisms, such as estuarine and coastal waters, and they tend to accumulate various particles, including viruses. However, the current knowledge on mollusk viruses is mainly centered on few pathogenic viruses, whereas a general view of bivalve-associated viromes is lacking. This study was designed to explore the viral abundance and diversity in bivalve mollusks using transcriptomic datasets. From analyzing RNA-seq data of 58 bivalve species, we have reconstructed 26 nearly complete and over 413 partial RNA virus genomes. Although 96.4% of the predicted viral proteins refer to new viruses, some sequences belong to viruses associated with bivalve species or other marine invertebrates. We considered short non-coding RNAs (sncRNA) and post-transcriptional modifications occurring specifically on viral RNAs as tools for virus host-assignment. We could not identify virus-derived small RNAs in sncRNA reads obtained from the oyster sample richest in viral reads. Single Nucleotide Polymorphism (SNP) analysis revealed 938 A-to-G substitutions occurring on the 26 identified RNA viruses, preferentially impacting the AA di-nucleotide motif. Under-representation analysis revealed that the AA motif is under-represented in these bivalve-associated viruses. These findings improve our understanding of bivalve viromes, and set the stage for targeted investigations on the specificity and dynamics of identified viruses.

## 1. Introduction

Viruses are the most abundant biological entity on the Earth, likely outnumbering bacteria and eukaryotic cells [1], with the oceans being the most likely richest reservoir of virus biodiversity [2]. The only constraint that viruses have is the need for a host for their replication, either to take advantage of the host replication machinery, or to hijack the genome to freely replicate as selfish genetic elements [3]. The evolutionary success of viruses is supported by highly dynamic genomes, which can undergo punctual changes or integration events that enable the circumvention of host immune defenses, the capture of new genes, and even host switching, among other events [4,5]. The frequent exchange of genetic material is evident in the highly variable sizes of viral genomes. While RNA viruses seem to have a ~32 kb size constraint [6], the genomes of DNA viruses can be uncommonly large, with the giant *Mimiviruses* genomes being in the order of megabases and far exceeding the few kilobases of circular single stranded DNA genomes of *cress viruses* [7,8]. The presence of an antiviral system in every living organism further supports the global distribution of viruses [9], although their biological roles go beyond pathogenicity [10]. In fact, viruses are responsible for selective pressures causing evolutionary transitions [11] as they drive the dynamics of host populations and interfere with biological invasions [12]. The gene flux from viruses to eukaryotic organisms is suggested to drive the long-term evolution of host genomes [13]. Conversely, the evolutionary pressure of host antiviral defenses shapes viral genomes in a never-ending arms race [14,15,16,17]. According to the sequence data currently available, the viruses identified so far represent a numerically insignificant portion of viral biodiversity, possibly no more than 1% of the extant viruses [1,18]. Thanks to an unprecedented level of sensitivity and accuracy, high-throughput sequencing (HTS) has become the gold standard for viral discovery and for advancements in the characterization of viral metagenomes [19,20], although most of the so-generated viral sequences remain “unclassified” due to uncertainty about authentic virus hosts. As viruses are mostly hidden in the host nucleic acids, and an unusually high sequencing coverage would be necessary to allow their detection, the current representations of the extant virosphere are fragmentary, and they crucially depend on sample preparation strategy, sequencing technology, and sequencing depth [21]. Nevertheless, DNA and RNA sequencing datasets often contain viral sequences, and committed analyses can provide snapshots of the viromes associated with a given organism [5,22].

Although it is steadily expanding, virus discovery and the study of antiviral immunity in invertebrates is biased towards a few model organisms and arthropods of economic and medical importance [23]. In this respect, highly speciose and ecologically important groups like mollusks, and the Lophotrochozoa more widely, remain largely unstudied, leaving huge taxonomic gaps in our knowledge. Since their initial diversification in the early Cambrian (Paleozoic Era), bivalves successfully colonized a variety of aquatic environments, from cold-water seas, to freshwater basins and deep anoxic vents, with some species showing an invasive behavior [24,25,26,27]. A number of bivalve species have been investigated for their peculiar adaptation strategies [28], innate immune systems [29], and bio-inspired applications [30] as well as for their use as models for human health [31]. Today, few bivalve genome drafts are available, whereas more than 2,100 transcriptomic datasets have been deposited in public databases (NCBI SRA archive, accessed in November 2018). So far, very few viruses of bivalve mollusks have been described, mainly those that have major negative economic impacts on farmed species. In particular, a herpesvirus (*Ostreid herpesvirus-1*, OsHV-1) associated with temperature-related oyster mortalities [32] has become a pressing issue for the production sector, and today, OsHV-1 and its variants are described in numerous studies [33,34,35,36,37,38]. Viruses belonging to the *Papovaviridae* and *Iridoviridae* families have been associated with bivalve diseases, whereas a few members of the *Togaviridae*, *Reoviridae*, *Birnaviridae*, and *Picornaviridae* virus families have been reported without evidence of associated disease [39,40]. Until the advent of HTS technologies, the identification of these viruses was mainly based on electron microscopy, and seldom validated by molecular studies [41].

Virome discovery through RNA HTS is challenging when applied to bivalve samples. According to the ability to detect minute quantities of viral nucleic acids, HTS also catches sequences that possibly derive from tissue surface contamination, or from the simple transit of another virus host in bivalve tissues [42,43]. The identification of giant viruses and human viruses in bivalve samples mainly accumulated in the gills and gut by filter-feeding [44,45,46], and the presence of an algal virus (Heterosigma akashiwo RNA virus-1) in the gills of both *Crassostrea gigas* and *Mytilus galloprovincialis* growing up in association [47], exemplify the importance of developing new approaches for assigning a virus to its authentic host. Ecological role and economic importance, peculiar genome features, differential susceptibility to pathogens, as well as their tendency to accumulate microbes highlights filter-feeding bivalves as fascinating models for virus–host interaction studies. The objective of this work was to explore the diversity and distribution of bivalve RNA viruses through the analysis of available RNA-seq samples. To do so, we performed an extensive analysis of the HTS transcriptome data of bivalves, we recovered bivalve-associated RNA viruses, and we traced their distribution over many bivalve RNA-seq samples. Moreover, we investigated how different RNA selection methods applied during library preparation can affect the performance of viral-oriented HTS analysis. Finally, we tested two different in silico approaches for assigning bivalve-associated viruses to their genuine hosts.

## 2. Materials and Methods

**Figure 1:**
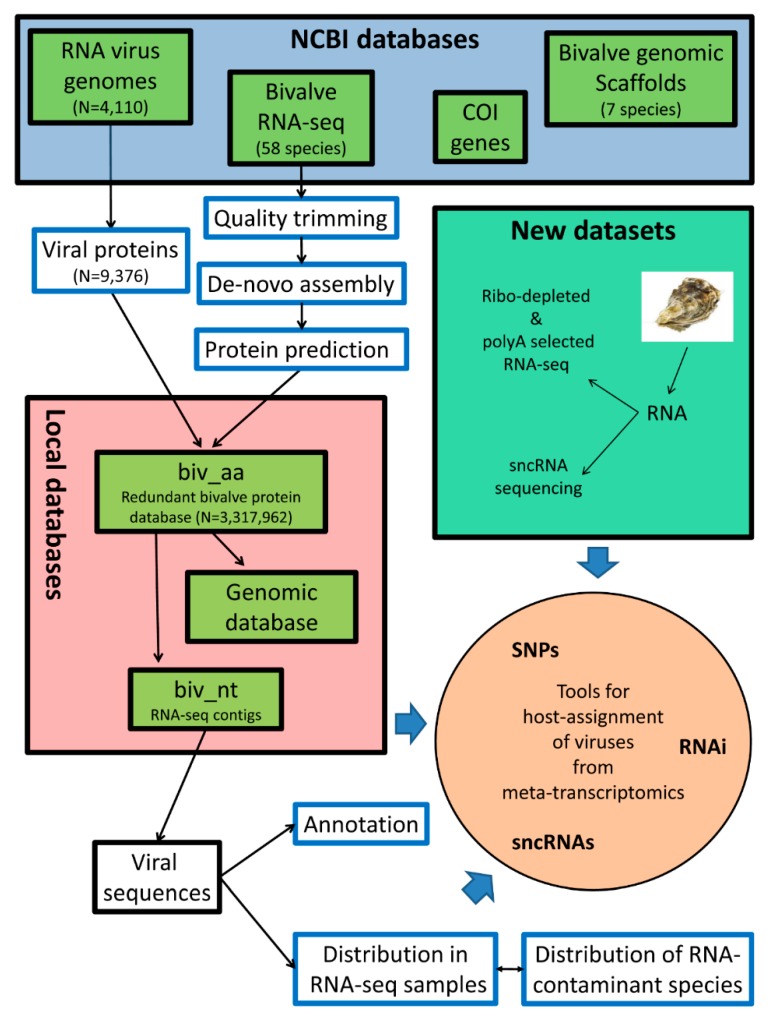
Summary of the analysis pipeline. The graph summarizes all of the steps that were used to extract viral sequences from bivalve RNA-seq datasets. Additional details are reported in corresponding sections of Materials and Methods.

The overall analysis pipeline is summarized in Figure 1, and it is detailed through the following paragraphs. The analyses are based either on the available RNA-seq datasets, or on newly produced data that we submitted to the NCBI SRA archive (the corresponding IDs are cited in the text and tables). 

### 2.1. Data Retrieval

Public sequence datasets were retrieved from NCBI databases in April 2017. A total of 7125 viral genomes, including 3008 RNA viruses, were downloaded from the NCBI Genome database. Additionally 1102 invertebrate-associated RNA virus genomes were downloaded from the NCBI nucleotide database [43,47] for a total of 4110 genomes of RNA viruses. RNA-seq samples referring to 58 bivalve species and four pooled bivalve or gastropod meta-transcriptomic samples were obtained from NCBI SRA archives. Genome drafts of five bivalve species (*Bathymodiolus platifrons*, *C. virginica*, *Mizuhopecten yessoensi*, *Modiolus philippinarum*, and *M. galloprovincialis*) were downloaded from the NCBI WGS database, while *C. gigas* and *Pinctada fucata* genomes were obtained from EnsembleMetazoa release35 and from [48], respectively. Supplementary Table S1 summarizes the sequence datasets used in this work. *Cytochrome C Oxidase Subunit I* (COI) sequences were downloaded from the NCBI nucleotide archive, and their redundancies were reduced using *cd-hit-est* [49], applying a cut-off of 95% similarity. In order to compare two RNA selection methods for their aptitudes of viral read recovery, we retrieved two RNA-seq datasets obtained from a single *C. gigas* sample (described elsewhere, SRR8237210 and SRR7636587 for polyA and ribo-depleted data, respectively).

### 2.2. Transcriptome de novo Assembly, ORF Prediction, and Protein Domain Mapping

RNA-seq reads were trimmed for the presence of adaptor sequences, and for quality, using TrimGalore! [50], allowing for a maximum of two ambiguous bases and a quality threshold of PHRED20. Trimmed reads were de novo assembled using CLC Genomic Workbench v.10 (Qiagen, Hilden, Germany), setting automatic word and bubble sizes, and a minimal contig length of 200 bp. The resulting contigs were subjected to open reading frame (ORF) prediction, using the transdecoder tool included in the Trinity suite [51], applying a minimal ORF length of 100 codons. HMMer v.3.1 [52] was used to identify the presence of conserved protein domains (Pfam-A models, v.29 [53], applying a cut-off *E*-value of 10^−5^.

### 2.3. Identification of Viral Sequences

A redundant BLAST database [54] was built, using the predicted proteins obtained from all of the transcriptomic assemblies of bivalve species. All the annotated ORFs encoded by 4110 RNA virus genomes were translated into amino acids, and the resulting 9376 protein sequences were used as blast queries against the bivalve database (blastp, cut-off *E*-value 10^−50^). Moreover, all the bivalve-derived protein sequences encoding a viral RNA-dependent RNA-polymerase (vRdRp) domain were selected. A vRdRp was identified by using six different PFAM Hidden Markov Models, corresponding to IDs: PF00680, PF00978, PF00998, PF02123, PF07925, and PF04197. Bivalve genome scaffolds were used to compose a genomic database to discriminate between host-encoded and viral sequences. Sequences identified from the redundant protein database, and from the search of the vRdRp domains, and showing no matches against bivalve genomes, were further processed to reduce the redundancy, applying a cut-off of 90% of similarity (*cd-hit*). The resulting protein sequences were used to recover the corresponding nucleotidic contigs from the initial transcriptome assemblies and they were considered as complete or partial genomes of RNA viruses. For the purposes of this paper, a viral genome was considered to be “nearly complete” if it was composed of a unique contig that was longer than 5 kb and encoding at least one complete ORF.

### 2.4. Distribution of Viruses among RNA-Seq Samples, Expression Analysis, and SNP Calling

The amount of reads mapping to the “nearly complete” viral genomes in selected RNA-seq samples was determined by stringent mapping of the trimmed reads on the viral genome sequences (0.9 both for length and similarity fraction, CLC mapper tool). For a selection of informative RNA-seq samples, the total read counts were used to calculate the percentage of reads mapping to each virus over the total numbers of reads of the sample, thus providing a comparison of the amount of viral RNA between RNA-seq samples that was not biased by different sequencing depths or read layouts. To obtain the expression profiles of selected oyster RNAi-related genes, 183 RNA-seq datasets (Supplementary Table S1) were mapped onto *C. gigas* gene models [55] and used to compute expression values such as transcripts per million (TPM) [56]. RNA-seq data were also used to call single-nucleotide variations (single-nucleotide polymorphisms, SNPs) across viral genomes. Specifically, to detect genuine SNPs, the trimmed reads were mapped onto the “nearly complete” viral genomes, setting 0.5 and 0.8 for the length and similarity fractions, respectively. A SNP was called if it was present in at least 1% of the locally aligned reads after using the following parameters: minimum average quality of the five surrounding bases, PHRED30; minimum required coverage, 50×; minimum required count, 5. The SNPs were annotated according to the neighbor base.

### 2.5. Estimation of the Contamination Levels of RNA-Seq Samples

To provide an estimation of the fraction of reads that were not related to the declared biological sample (as indicated in the SRA details), we mapped the RNA-seq reads onto a collection of 205,357 non-redundant COI sequences. Reads were mapped applying a similarity fraction of 0.8, over 0.8 of the read length (CLC mapper tool) and the TPM values were computed. Similarly, to estimate the presence of known viruses among the RNA-seq datasets, the amount of reads mapping to the 7125 virus genomes obtained from NCBI was also computed (in this case, by applying 0.9 and 0.9 for the length and similarity fractions, respectively).

### 2.6. Small RNA Sequencing and Reads Analysis

The fraction of small RNAs (<200 bp) of the *C. gigas* sample that was used to prepare the paired polyA and ribo-depleted libraries were extracted using the Mirvana kit (Thermofisher, Waltham, MA, USA). RNA was quantified by using a Qubit fluorimeter instrument, and the RNA size profile was determined with an Agilent small RNA chip (Agilent, Santa Clara, CA, USA). Library preparation and sequencing (PE150) was outsourced and carried out on an HiSeq Illumina platform (Admera Health, New York, NY, USA), and submitted to the NCBI SRA archive, under the accession ID SRR8587800. The paired reads were trimmed for quality, and for the presence of adaptors, as described for mRNA reads, and the correctly paired reads were joined into fragments. The resulting clean fragments, in a length range of 15–50 nt, were used for the detection of viral-derived small RNAs (vsRNAs) by direct mapping on the identified viral contigs or by using the VirusDetect pipeline [57]. To discriminate between genuine vsRNAs versus RNA degradation products, we correlated the number of mapped sRNA reads with the viral expression levels. 

### 2.7. Analysis of Viral Genome Editing

The genomes of the RNA viruses retrieved from NCBI were analyzed for the distribution of the frequency of di-nucleotides as hallmarks of the virus genome fitness (i.e., adaptive genome changes). To look for these adaptive genome changes, we used the cytidine deaminase under-representation reporter (CDUR) [58]. Given the user-defined motifs and an input coding sequence, CDUR effectively utilizes a permutation test to determine whether the given motif is significantly depleted in the input sequence than one would expect by chance (details below). The two main metrics that are analyzed are: 1. the “below” metric, which determines whether the number of occurrences of a motif is significantly fewer than expected, and 2. the “repTrFrac” metric, which determines the ratio of motifs that would incur a non-synonymous transition mutation, against the total number of those motifs in the sequence, which is significantly fewer than expected. Both of these metrics are determined by shuffling the coding sequence at the third position of each codons, so that the underlying amino acid sequence is unchanged. We chose this method of shuffling as it also preserves the GC content of the input sequence, as changing the GC content has been reported to yield biased results [14]. This shuffling is repeated 1000 times; in each shuffled sequence, we counted the number of user-defined motifs (“below” metric), as well as the ratio of nonsynonymous transition mutations that occur at those motifs, compared to the number of motifs (“repTrFrac” metric). In both cases, we determined the percentage of shuffled sequences with fewer motif counts and repTrFrac counts than that of the input, to yield a statistical *p*-value. A sequence with a *p*-value of <0.05 is said to be under-represented in that motif metric, whereas a *p*-value of >0.95 is said to be over-represented in that motif metric (see Figure 2 in [58]).

A particularly interesting case is when, for a given motif, a sequence is under-represented in the “below” metric, and is over-represented in the “repTrFrac” metric for that motif. This suggests that this sequence has maximally tried to reduce the number of occurrences of that motif, as any further reduction would result in amino acid changes, which may negatively impact that coding sequence. Recent studies have shown that certain gammaherpesvirus may be under such pressures [59]. In this case, the sequence is considered to have attained maximal under-representation. We performed CDUR analysis on 3872 RNA viral genomes with a known host obtained from NCBI (Supplementary Table S1), as well as on the newly recovered “nearly complete viral genomes” presented in this paper.

### 2.8. Phylogenetic Analysis

Protein sequences referring to vRdRP domains were aligned using MUSCLE [60], and trees were generated with MEGA 6 [61], using neighbor-joining clustering methods with 100 bootstrap replicates. The phylogenetic tree was uploaded to the iTOL server for easier visualization [62].

## 3. Results

We produced transcriptomic assemblies of 58 bivalve species, and we used all of the predicted proteins to produce a redundant BLAST database, including more than 3 million entries (hereinafter called biv_aa). To identify the putative virus sequences associated with the analyzed RNA-seq samples from different bivalve species, we queried biv_aa with 9376 protein sequences belonging to 4110 known RNA virus genomes (Supplementary Table S1). We extracted additional viral sequences from the same database by searching all six available PFAM domains of viral *RNA-dependent RNA polymerase* (vRdRp). In the absence of a conserved viral gene, we used these domains to identify RNA viruses, since vRdRp is needed for the transcription of the viral genome during productive virus replication [63,64]. For the BLAST searches, we applied a conservative *E*-value of 10^−50^, and to further limit false positive results, we discharged the BLAST matches with less than five hits. Moreover, we screened all of the positive hits against a database composed of available genomic scaffolds of bivalve species, to remove genomically encoded sequences (Supplementary Table S1). As a result, 708 biv_aa entries showed a genuine similarity to viral sequences, and the conserved domains included in these proteins further sustained their viral origin, since we found 253 vRdRp, 80 *CRPV capsid protein like*, 73 RNA *helicase*, 78 *Dicistroviridae minor capsid protein*, and 69 *Picornavirus capsid protein* domains (Supplementary Table S2). The removal of similar sequences (>90% of similarity) resulted in 413 unique sequences. Most of the redundant sequences were found in transcriptomes of the same bivalve species, either in RNA-seq samples originating from the same geographical location, as in the case of *M. galloprovincialis* or *C. gigas* samples from Goro (Italy), or obtained from geographically unrelated samples of the same species, although few exceptions are present, and they are discussed below. Despite most sequences being retrieved by BLAST searches using viral sequences as queries, they showed a limited similarity to known viruses (Supplementary Table S2). We could confidently assign only 15 sequences (3.6% of the total) to 11 known viruses (BLASTn with an *E*-value lower than 10^−100^ and identity >95%), namely, six bivalve-associated RNA viruses from the lagoon of Goro (Italy), two viruses associated with marine invertebrates from China, plus three other RNA viruses, the algal *Heterosigma akashiwo RNA virus*, the plant virus *Zygocactus virus X*, and the *Sacbrood virus* (Supplementary Table S2).

### 3.1. Effect of RNA-Seq Library Preparation Protocols on the Detection of Viral Sequences

In order to evaluate the effect of the RNA selection method applied during library preparation on the recovery of viral reads, we analyzed two different datasets, each of them derived from a single biological sample by using alternative RNA selection approaches: polyA RNA selection or ribosomal RNA depletion. The first dataset was prepared specifically for viral meta-transcriptomic analysis, starting from two biological samples (named “mix of bivalves”, sample ID: SAMN04625952 and “mix of gastropods”, sample ID: SAMN04625958 [43]). We analyzed a second dataset obtained from a single *C. gigas* specimen naturally infected with OsHV-1, using the same two RNA selection methods (sample ID: SAMN09760011). The analysis of the four meta-transcriptomic samples showed that, despite a considerable variability in the numbers of raw reads, the assembled contigs, as well as the number of predicted proteins yielded somewhat comparable values, except for SRR3401755, for which only few proteins could be predicted. For these datasets, polyA-selection allowed for the recovery of a higher ratio of viral to total proteins, particularly for the bivalve samples (Table 1). On the contrary, the analysis of the oyster dataset clearly showed the opposite trend in terms of viral read recovery, since we identified 46 contigs encoding a vRdRP domain in the ribo-depleted sample, compared to 10 in the polyA selected one. Although five out of the 10 polyA viral contigs were also found in the ribo-depleted dataset, the longer contig was always generated from the ribo-depleted dataset.

### 3.2. Identification of “Nearly Complete” Viral Genomes

As mentioned above, we putatively identified 413 viral protein sequences in 364 nucleotidic contigs, indicating that some contigs included more than one viral ORF. Theoretically, each contig can be considered as a viral genome, but if we compare their average length (1.39 kb) with the median lengths of the known RNA virus genomes (4.8 kb), a realistic assumption is that most of these represent incomplete genomes. For the purpose of this paper, we considered “nearly full-length viral genomes” as only being contigs that are longer than 5 kb and encoding at least one complete ORF. Therefore, we identified 26 contigs ranging in length between 5.4 and 9.7 kb as being “nearly complete viral genomes”, with 12 contigs encoding two ORFs corresponding to one replicative and one structural protein, while the other contigs (14) encoded a single ORF. These “nearly complete” viruses were named according to the species from which they were assembled (for instance, viruses identified in RNA-seq samples rich in viral sequences and referring to *C. gigas*, *M. galloprovincialis*, *Ruditapes philippinarum*, and *M. edulis*), whereas the unique viral contig found in the *Elliptio complanata* transcriptome (Table 2) was a complete viral genome (Elicom_virus1, 7106 nt). Notably, five nearly complete viral genomes sequences were identified in our ribo-depleted *C. gigas* RNA-seq sample. A total of 10 nearly complete viral genomes sequences could be assigned to a known virus, while other eight other sequences displayed an intermediate/low similarity to known viral sequences, and eight other different sequences referred to completely unknown viruses (the latter being associated with RNA-seq samples of *C. gigas* (4), *E. complanata* (1), *M. galloprovincialis* (1), *Mizuhopecten yessoensis* (1) and *R. philippinarum* (1)). In three viral genomes (*Bivalve RNA virus G1, Rudphi virus 4*, and *Heterosigma akashiwo RNA virus*-1) we could identify a polyA tail at the 3’ end of the sequence (Supplementary Figure S1). New sequences or sequences not fully matching the known viral genomes have been deposited in the NCBI database, and the accession IDs are reported in Table 2.

Subsequently, we evaluated the distribution of these 26 viruses in 226 RNA-seq samples, referring to their putative host species. Since the initial removal of redundant viral proteins suggested that some of these viruses are distributed over RNA-seq samples of multiple species, or they originated from samples that were possibly contaminated by pathogen-associated (e.g., *Perkinsus spp*.) RNAs, we included additional 24 RNA-seq samples in the distribution analysis, for a total of 250 datasets (Supplementary Table S3).

**Figure 2:**
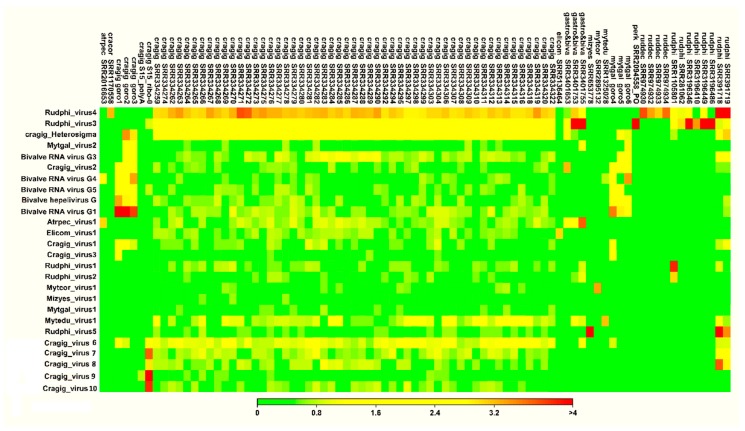
Coverage heat map for the 26 bivalve-associated viruses over 82 RNA-seq samples with at least 1000 viral reads. Data are reported as log_10_, as depicted in the colored scale. Raw data are reported in Supplementary Table S3.

More than eight million reads were mapped onto the 26 viral genomes, with 862,949 and 7,092,869 reads that matched the *Rudphi_virus3* and *Rudphi_virus4* genomes, respectively. Twenty-two viral genomes were covered by at least 1000 reads, and 82 out of 250 RNA-seq samples included more than 1000 viral reads, and for this reason, they were selected for further consideration (Supplementary Table S3). In these 82 samples, the fraction of viral reads over the total ones per single virus usually did not exceed 1‰, except for *Rudphi_virus4*, which was covered by 30‰ of total reads for a larval *R. philippinarum* RNA-seq sample, and *Rudphi_virus3*, which reached 6‰ in one gastropod meta-transcriptomic sample (Table 2, and Supplementary Table S3). Few viruses showed a distribution over samples of different bivalves, e.g., *Rudphi_virus4* (present in *R. decussatus*, *R. philippinarum*, *C. cortenzinesis*, *C. gigas*, and *M. edulis* samples) and *Bivalve RNA virus G4* (present in *C. gigas*, *M. galloprovincialis*, and *Atrina pectinata*, Figure 2). Moreover, the occurrences of *Rudphi_virus4* and *Rudphi_virus3* go beyond bivalve species, since we traced them both in metagenomic gastropod samples. Since *Rudphi_virus3* originated from a *Perkinsus*-infected sample of *R. philippinarum*, and it was traced in 12 clam datasets, we further investigated the presence of this virus in the publicly available *Perkinsus* transcriptome data (11 RNA-seq samples, Supplementary Table S1). As a result, some reads (3.9‰) of a sample of *Perkinsus olseni* trophozoites exposed to clam plasma (SRR2094558) were mapped to this virus, whereas, only 22 viral reads (<0.00001‰) were detected in the paired control (Supplementary Table S3). In contrast, other viruses were associated to the unique RNA sample, for instance, *Elicom_virus1*, *Rudphi_virus5*, and *Mytedu_virus1* (Figure 2 and Supplementary Table S3).

### 3.3. Evaluation of Contaminant RNAs in RNA-Seq Samples

We mapped the reads of 16 selected RNA-seq samples to a collection of COI gene sequences, namely, *C. gigas* S15 (Ribo-0 and polyA), a “mix of gastropods” (Ribo-0 and polyA), one *P. olseni*, four *R. decussatus*, and seven *R. philippinarum* samples, since they included reads of multi-host viruses (Figure 2). We calculated the fraction of reads mapping to each COI entry over the total reads that mapped onto the whole COI dataset, and we used it as a tool to evaluate the contribution of biological contaminants in each RNA-seq dataset. As result, 159 COI entries showed at least 0.01% of mapped reads (Supplementary Table S4). Obviously, the first COI entry of each sample corresponded to the sequenced biological sample, thus confirming that the gastropod mix samples were composed of multiple species. For some samples, we observed additional COI entries with lower percentages, as in the two *R. philippinarum* samples with the highest numbers of viral reads (SRR391718-19), where we detected several contaminant species (56 and 57 COI entries with at least 1% of mapped reads, respectively). We noted the presence of a known bivalve-associated tunicate (*Diplosoma listerianum*) in 10 of the tested RNA-seq samples. Although the *D. listerianum* COI value is equal to 100% in the *P. olseni* sample, due to the absence of the Alveolata entries in the COI dataset, our analysis confirmed the absence of clam RNAs in the *Perkinsus* samples, including a high level of *Rudphi_virus3*. Intriguingly, both the Ribo-0 and polyA S15 datasets showed a low contamination of *Lacconectus peguensis* (Coleoptera).

### 3.4. Tools for the Host-Assignment of Bivalve-Associated Viruses

Our analysis further demonstrated that most of the transcriptome-derived viruses could be only tentatively assigned to a specific host, due to their occurrence in samples of even phylogenetically distant species. Under this context, the application of coverage cut-offs appeared to be an unreliable approach for host-assignment. Therefore, we investigated the feasibility of two alternative approaches for the host-assignment of bivalve-associated viruses obtained from transcriptomic data, as follows.

**Figure 3:**
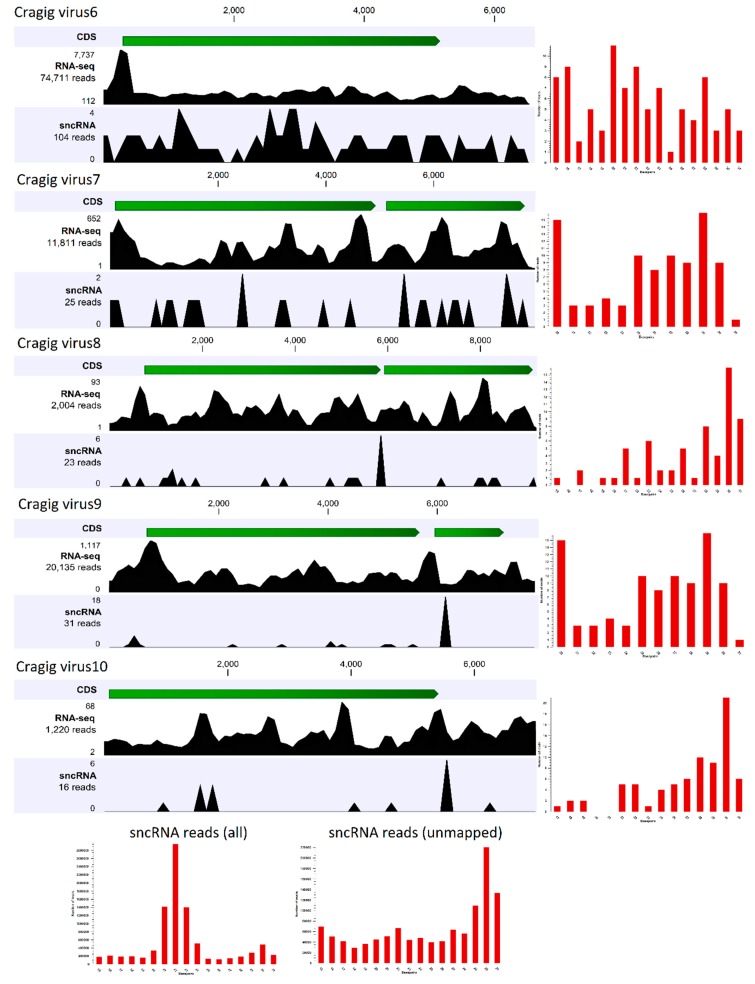
Small non-coding RNA (sncRNA) reads analysis. The RNA-seq and sncRNA read distribution for each of the five “nearly complete viral genomes” reconstructed from the ribo-depleted oyster RNA-seq data are shown. The open reading frames (ORFs) for each virus are shown in green, while the number of mapped reads are reported on the left. The histograms on the right represent the size distributions of the mapped sncRNA reads (in the range of 15–31 nt). The bottom histograms show the size distribution for the whole library (left), and for the reads that did not match the oyster genome (right).

The first approach investigates the presence of virus-derived RNAi products (vsRNAs), and it is used to reconstruct full-length genomes of viruses infecting arthropods [65,66,67]. Since the antiviral role of the RNAi system of bivalves has never been demonstrated, we firstly investigated the expression patterns of selected RNAi-related genes (*DICER*, *DROSHA*, *ARGONAUTE*, *PIWI*, and *RNA-dependent RNA polymerase*) in 184 *C. gigas* RNA-seq samples, including some samples that were very rich in viral reads (Supplementary Table S1), to correlate the gene expression values with the presence of actively transcribing RNA and DNA viruses (Supplementary Table S5). We showed that RNAi-related genes are mostly expressed in the early developmental stages of oyster, when two PIWI and one Argonaute transcript showed remarkable expression levels (Supplementary Table S5, panel A), and PIWI1 was preferentially expressed in gonads (Supplementary Table S5, panel C). Apart from these samples, we reported a considerable expression of PIWI1 in three oyster gill samples, and in an additional sample referring to adductor muscles (SRR334286). While the latter result is difficult to explain, the expression of PIWI1 in the oyster gill samples from Goro (Italy) correlated with the presence of RNA viruses (see Figure 2, Supplementary Tables S3 and S5). Although at lower expression levels we reported that one *RNA-dependent RNA polymerase* transcript (EKC38952), belonging to a gene family typically expressed in the digestive gland, showed considerable expression levels in a few other samples, namely two out of three biological replicates of oysters infected with OsHV-1 (12 hours after infection, gills) and a spat sample highly infected by the same virus (Supplementary Table S5, panel B; sample G1). Taken together, these results provide limited evidence for an active role of some components of the RNAi pathway during viral infections in oyster. To further investigate the functionality of RNAi as antiviral system, we sequenced the fraction of small RNAs of the *C. gigas* sample used for library comparison, and found a high number of viral reads belonging both to DNA and RNA viruses (see Figure 2). Small non-coding RNA (sncRNA) sequencing yielded 10.1 million clean fragments in a length range of 15–50 nt. A total of 22,587 sncRNA reads matched the viral contigs identified in this sample, plus the OsHV-1 genome. However, we observed a positive correlation between the expressions of viral genes (using both Ribo-0 and polyA datasets) and the number of sncRNA reads that matched these ORFs (r^2^ of 0.994 and 0.996, respectively), suggesting that the reads mostly originated from RNA degradation products, instead of being genuine vsRNAs. We further analyzed the coverage of the sncRNA reads along the five “nearly complete viral genomes” originating from the S15 oyster ribo-depleted data. To do this, we mapped to the viral genomes the sncRNA reads that did not match to the oyster genome (1.436 M reads), and we calculated the size profiles of each of the mapped subsets (Figure 3). Notably, comparing the size profile of the whole sncRNA library with the profile of the sncRNA reads that did not match to the oyster genome, we showed that *C. gigas* sncRNA reads peaked at 21 nt (microRNAs), whereas the unmapped sncRNA reads peaked at 30 nt, indicative of their Piwi-interacting RNA (piRNA) nature. However, only 199 sncRNA reads mapped to one of the five viral genomes reconstructed by using the paired Ribo-depleted RNA-seq reads, and the size profiles showed a low enrichment of 29–30 nt reads with a distribution over the whole viral genome (Figure 3).

Moreover, we subjected the sncRNA reads to VirusDetect, a bioinformatics pipeline that is designed for the identification and reconstruction of viral genomes starting from short reads [57]. Although 169,970 sncRNA reads could be aligned to the viral reference database, and the tool could assemble 40 contigs, the 20–22 nt enrichment fraction was always low, and it did not support their vsRNA nature. According to the presence of numerous OsHV-1 reads in the paired RNA-seq data (the polyA and Ribo-0 datasets), VirusDetect identified several matches to the OsHV-1 genomes, but again, with a low 20–22 nt enrichment fraction.

**Figure 4:**
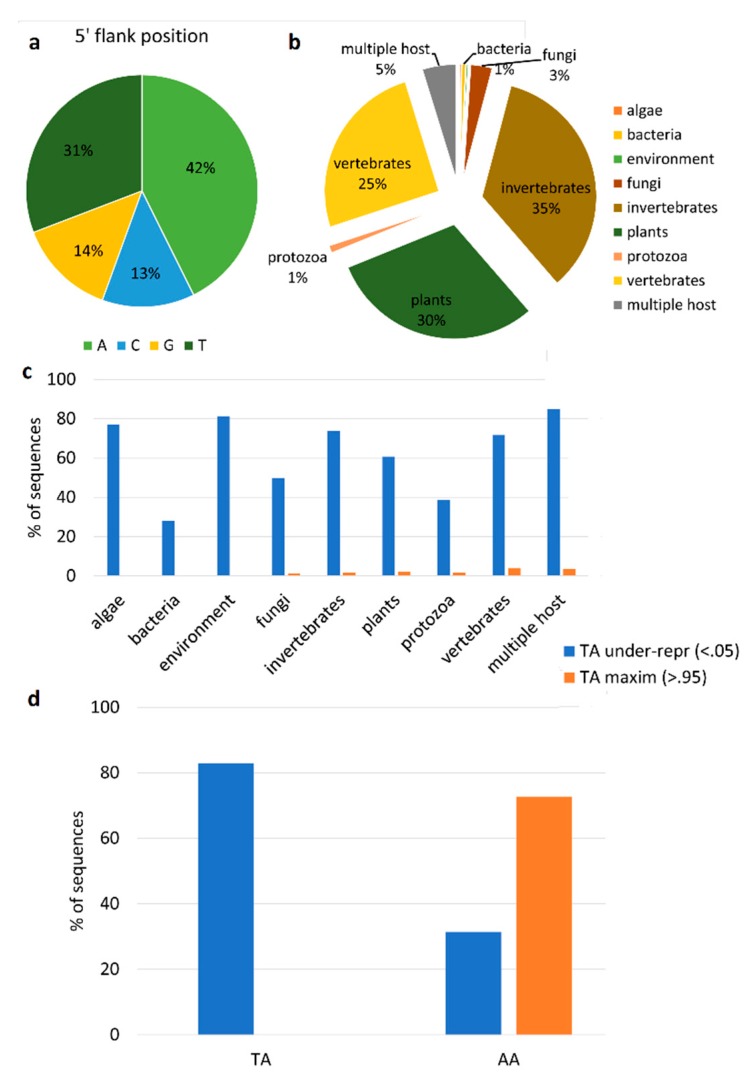
Analysis of virus editing. (**a**). Distribution of the flanking base of ADAR-compatible SNPs. (**b**). Host distribution of the 3872 viral genomes used as a training set. (**c**). Percentage of viral sequences (*N* = 3872) showing statistically significant TA under-representation (<0.05) and maximization (>0.95), divided per host class. (**d**). Percentage of TA and AA under-representation (<0.05) and maximization (>0.95), measured on the coding sequences of the nearly complete viral genomes reported in this paper.

The second approach that we tested leveraged on the identification of single-nucleotide modifications (SNPs) occurring specifically on viral transcripts produced by the action of host enzymes acting as antiviral defenses. Therefore, we attempted to select and count the subset of total SNPs generated by the host double-stranded RNA (dsRNA) editor enzyme *adenosine deaminase acting on dsRNA* (ADAR), which is assumed to specifically modify viral dsRNAs through A-to-I editing [68]. For each of the 26 viruses, we selected the RNA-seq sample with the higher number of reads, and we called these low-frequency SNPs; among the identified SNPs, we selected the ADAR-compatible ones (A-to-G). We identified 7569 SNPs located on viral coding sequences, and we classified 938 of them as being ADAR-compatible. Considering the 5’ position, we showed that 31% of the selected SNPs had an adenine at the flanking position, while 42% had a thymine (Figure 4a). Also, we searched for the evolutionary footprint of the action of ADAR on viral genomes in parallel. To do this, we used the CDUR tool [58] (see Materials and Methods) to determine under- or over-representation of a motif in a given sequence. Firstly, we used a training set of 3872 genomes of RNA viruses with a known host (Figure 4b and Supplementary Table S1). By analyzing the WA (W = A/T), AA, CA, GA, and TA motifs, the CDUR analysis showed that the TA motif is under-represented in 62.7% of the analyzed ORFs, while the AA, GA, and CA motifs are under-represented in 32.9, 8.1, and 1.5% of ORFs, respectively. Intriguingly, 4% of TA-under-represented ORFs maximized this under-representation, since additional variations will cause non-synonymous SNPs. Although we have to take into consideration that the viral representatives of each of the host classes are variable (Figure 4b), by linking the under-representation values with the viral host, we showed that most (>70%) of the algae, invertebrate, and vertebrate viruses reduced the TA motifs in their coding regions, while we observed moderate percentages (50–60%) for fungal and plant viruses, and lower percentages for bacterial and protozoa viruses (Figure 4c). Accordingly, the sequences with a maximization of the TA reduction were only a small fraction of the ones for fungal, plant, invertebrate, protozoa and vertebrate viruses (Figure 4c). Subsequently, we used the CDUR package to investigate the under-representation of the motif in the ORFs of the nearly complete RNA virus genomes described in this paper (Figure 4d). Consistent with the previous results, only the TA and AA motifs were statistically significantly under-represented. However, we did not observe ORFs with maximized TA reductions, while eight out of 11 ORFs showing AA being under-represented, significantly maximized the AA motif reduction (Figure 4d).

To better contextualize our results, and to assign the 26 “nearly complete viral genomes” to a viral group, we attempted a phylogenetic analysis based on the regions corresponding to the vRdRP domains (the phylogenetic tree can be visualized at [69], or as Supplementary Data S1). The phylogenetic tree obtained by the comparison of 2019 sequences of viral origins showed poor bootstrap support for most of the nodes, due to the high heterogenicity of the vRdDP sequences. Several of the sequences of the 26 viruses reported herein clustered with picoRNA-like viruses obtained from meta-transcriptomic surveys of mollusk species [43]. These viruses included *Mytedu virus1*, *Myzyes virus1*, *Cragig virus1*, *Cragig virus2*, *Cragig virus3*, *Cragig virus6*, *Rudphi virus4*, *Rudphi virus5*, and *Bivalve RNA virus G3*. *Cragig virus 10* showed similarities with *Bivalve hepelivirus G* (herpes-like viral family, as defined by [43]). Although *Cragig virus7* also clustered in a group of picoRNA-like viruses, it appeared to be separated from the other marine picoRNA-like sequences. Similarly, *Cragig virus8* and *Cragig virus9* formed a cluster including picoRNA-like viruses and one diatom virus (*Chaetoceros socialis f. radians RNA virus1*). None of our viruses grouped in clusters were characterized by the presence of abundant vertebrate viruses, while *Mytcor virus1* was grouped with plant viruses, supporting its BLASY similarity to *Pitaya virus X*.

## 4. Discussion

Viruses can infect almost every living organism, and viral nucleic acids, either DNA or RNA, are often found when the host sequences are analyzed, making host RNA-seq samples suitable targets for viral discovery [21,70]. In this study, we analyzed bivalve RNA-seq data, and recovered both partial and complete RNA virus genomes, exploiting them to investigate the limits of meta-transcriptomics approaches oriented to viral discovery in bivalves. We identified 413 unique sequences of viral origin, most of them showing a limited similarity with known viruses, demonstrating that bivalve RNA sequencing allows for the identification of viral sequences. These included 26 nearly complete viral genomes. Although the factors that mostly influence the number of viral reads in an RNA-seq sample seem to be the strategies used for sample collection (e.g., the inclusion of water present in the shell cavity), we showed that the RNA selection method used during library preparation also contributed to the recovery of viral reads. For the samples prepared specifically for viral meta-transcriptomics, it was demonstrated that it is possible to recover multiple RNA virus genomes from single samples [43], while analyzing RNA-seq data (polyA-selected) originally designed for host expression analysis, we could identify at most, one complete viral genome per sample, over multiple (partial) viral genomes. Analyzing available meta-transcriptomic data, we found that polyA-enrichment is somewhat more effective than ribosomal-depletion in term of viral read recovery. However, we demonstrated that ribo-depletion is capable of higher performance, since we could reconstruct five nearly complete viral genomes from a RNA sample prepared for oyster expression survey. Arguably, polyA-selection would bias the virus sequence identification in the case of polyadenylated viral genomes (e.g., *Picornavirales*), although we could find evidence of the presence of polyA-tails only in three out of 26 viruses. Overall, our result strongly enforced the use of ribo-depletion for the preparation of RNA-seq libraries targeting viral discovery.

In agreement with a recent study reporting a wide host distribution of invertebrate viruses [43], we traced six out of 26 viruses in RNA-seq samples of different bivalve species, and we reported three bivalve-associated viruses that were very similar to viruses identified from gastropod or sponge meta-transcriptomics data [70,71]. The presence of identical viruses in different bivalve species, or even in phylogenetically distant invertebrates has two possible explanations: either these viruses infect a broad-range of animals, or the species hosting these viruses is shared by different (marine) animals. In support of the first hypothesis, even if invertebrates (arthropods in particular) are rich in viruses [72], strong evidence for host–virus co-evolution was rarely reported [17], and host jumping seems to be common for invertebrate viruses [5]. These attributes are in agreement with the new concepts of RNA virus phylogenesis that are inferred by viral metagenomics, suggesting extensive horizontal virus transfer events and a broad host range for protostome viruses [73]. The second hypothesis, i.e., that these viruses are hosted by an organism that is common in the marine environment, may be the easiest explanation for the presence of identical viruses in samples of different species, and can be further supported by the filter-feeding activity of bivalves. In fact, given the functions that are exerted by the gut and gills (the latter tissue is commonly used for RNA-seq experiments), contamination by RNA originating from waterborne bacteria, fungi, microalgae, or even microeukaryotes, is common in bivalve RNA-seq samples. This situation is well-depicted by one of the complete genomes that we recovered, the algal virus *Heterosigma akashiwo RNA virus*, which we traced in RNA-seq samples of co-cultured *C. gigas* and *M. galloprovincialis*, and even in an unrelated *R. philippinarum* sample. In this study, we exploited the COI reads to identify possible co-occurring organisms of the RNA-seq samples rich in viral reads. Although COI is not a universal gene marker, such an analysis can provide an immediate view of the purity of the samples [71]. In our study, the COI analysis did not identify a contaminant organism that completely matched the distributions of multi-species viruses. The contamination with *D. listerianum* RNA present in several bivalve RNA-seq samples confirmed the wide distribution of this fouling tunicate, but it could represent only a partial explanation for the multi-species distribution of *Rudphi_virus3*. The *R. philippinarum* sample, including 3% of *Rudphi_virus4* reads was shown to be heavily contaminated by this tunicate, and by other non-bivalve species, but none of these species correlated with the distribution of this virus.

The determination of the host of meta-transcriptomics-derived viruses is likely one of the main challenges of viromics based on high-throughput data [23]. At the tissue level, both Transmission Electron Microscope (TEM) imaging of viral particles and in situ hybridization techniques are suitable to confirm host assignment only if infection intensity is sufficiently high, but they are unfeasible in the case of a very large number of samples, or for already-sequenced RNA-seq samples, such as the ones that we analyzed. Therefore, we investigated the feasibility of two alternative approaches that, exploiting the same RNA-seq samples used for viral discovery, can provide evidence that is useful for host-assigning bivalve-associated viruses. RNAi is used as an antiviral defense in plants, insects and nematodes, where efficient RNAi processing of viral genomes into virus-derived small RNAs (vsRNAs) perfectly matching to the original genome activates the RNA-induced silencing complex (RISC), which in turn catalytically marks viral sequences for degradation [74]. Differently from immuno-recognition mechanisms based on antibodies, RNAi is not impacted by viral mutations, and in *C. elegans*, it functions at nano-molar concentrations thanks to the amplification of vsRNA signals using RNA-dependent RNA polymerase [75]. Recently, an RdRP-independent mode of RNAi amplification has been reported in *Drosophila*, although the underpinning genetic mechanism is still unknown [76]. At least in worms, RNAi-based antiviral immunity can be generationally transmitted, and provide a kind of epigenetic immune-memory [77] that recalls the prokaryotic CRISPR-Cas-based adaptive immunity [78]. Recently, the identification of vsRNAs in a molluscan gastropod (*Nucella lapillus*) opens intriguing questions about the phylogenetic distribution of the antiviral defense system, and about the mechanism itself [71]. In insects, the analysis of the fraction of vsRNAs among sncRNA datasets allowed for an unbiased reconstruction of pathogenic viruses [65,66,67] but, although bivalve antiviral immunity partially resembles that of arthropods [79], the antiviral role of RNAi has never been directly demonstrated [80]. Our analysis suggested that RNAi exerted limited importance in antiviral defense in bivalves, since even if we showed that *C. gigas* PIWI1 is induced in RNA-seq samples containing RNA viruses and a more limited induction of one oyster RDR gene was consistent with the active transcription of OsHV-1, we were not able to clearly identify vsRNAs among sncRNA reads obtained from the same oyster sample, including abundant reads of RNA viruses and of OsHV-1 (dsDNA virus). Considering all of the viral contigs obtained from this RNA-seq dataset, our analyses strongly suggest that the sncRNA reads that mapped on these viral contigs are due to RNA degradation. Differently, looking only at the five complete viral genomes, we could not exclude that weak RNAi activity generated few vsRNAs. Improving the power of our analysis by increasing the coverage or by using chemical treatments to specifically enrich the sncRNA fraction [71], it would be possible to detect genuine vsRNAs, even in bivalves. In particular, the size profiles that we reported for the putative vsRNAs seemed to be biased through a non-Dicer production mechanism [65,71].

In vertebrates, the antiviral role of RNAi is superseded by the interferon pathway, which through the activation of interferon-stimulated genes (ISGs), promotes the recognition of viral-derived products and inhibits viral propagation. Among ISGs, powerful sequence editors like ADAR and *apolipoprotein B mRNA editing enzyme, catalytic polypeptide-like* (APOBEC), enzymatically mutate viral transcripts and genomes [81,82] by acting on target sites that are highly conserved throughout the metazoan evolution [14,83,84,85]. To counteract these host-mediated editing mechanisms, some viral genomes have evolved to reduce the frequency of sites that are more vulnerable to targeting by the host immune system [58,86,87]. Surprisingly, we demonstrated a diffuse under-representation of the “TA” motif in most of the known RNA viruses, although only few of them maximized this reduction, and we could not link this result to a specific class of hosts. A similar trend of TA under-representation was also present in the 26 viruses described herein, but for these viruses, we showed that there was a tendency to maximize the reduction of the AA motif. Similar to the 26 viruses reported herein, a similar trend characterizes, among others, the *Antarctic picorna-like virus 1* and 3, *Acute bee paralysis virus* and *Aphid lethal paralysis virus*, which represented *Picornavirales* with a phylogenetic vicinity with some bivalve-derived viruses [47]. SNP analysis did not highlight a predominant fraction of ADAR-compatible variations over the total SNPs in the 26 viruses. Arguably, CDUR analysis only determined under-/over-representation, and the possible role of ADAR as source of these shifts should be confirmed by dedicated experiments.

Overall, This study underlines the heterogeneity and variability of RNA viruses that are associated with marine mollusks, and the limited data that is available on environmental RNA viruses. While the simultaneous analysis of viral products, antiviral host defense processes, and products in the RNA-seq samples could support host assignment, this alone is not enough when dealing with suspension-feeders that are able to accumulate environmental microbes, and their viral symbionts. Given the growing body of knowledge on the role of viruses in host fitness, targeted investigations aimed at unraveling the diversity of “genuine” bivalve viruses are needed for a better understanding of factors affecting the health and well-being of these ecologically- and economically-important species.

## Figures and Tables

**Table 1:** Assembly statistics of four viral-metagenomic samples. SRA and sample IDs, RNA selection methods, number of reads in million, and the number of assembled contigs and predicted proteins are reported. The number of viral RNA-dependent-RNA-polymerase (vRdRp) domains, the ratio of viral protein to total proteins, as well as the number of complete viral genomes, are also indicated.

SRA ID	Sample ID	RNA Selection Method	No. of Reads (M)	No. Of Assembled Contig	No. of Predicted Protein	No. of vRdRp	Ratio of Viral Proteins	No. of Complete Genomes *
SRR3401648	SAMN04625952	Ribo-depletion	99.7	96,102	45,343	30	0.00007	2
SRR3401653		polyA-selection	58.3	120,399	38,498	129	0.00034	9
SRR3401753	SAMN04625958	Ribo-depletion	47.9	180,272	48,687	43	0.00009	6
SRR3401755		polyA-selection	60.3	105,611	14,661	54	0.00037	4
SRR7637587	SAMN09760011	Ribo-depletion	54.1	156,166	41,785	46	0.00011	5
SRR8237210		polyA-selection	52.0	93,172	40,301	10	0.00002	0

* additional details on the complete viral genomes are reported in Table 2.

**Table 2:** Summary of the 26 nearly complete viral genomes. Bivalve species, total number of viral sequences, non-redundant (nr) sequences, and number of “nearly complete viral genomes” are reported. Sequenced tissue and geographical origins of the RNA samples, library type, virus name, and virus distribution in transcriptomes of other bivalves and virus coverage are also reported (as is the total number of viral reads, and as a percentage over the total reads). The BLAST similarities are reported with the *E*-values, NCBI ID, and the description and percentage of identity.

Species	Viral Sequences			Tissue	Geographic Origin	Library Type	Virus Name	Virus Distribution among Bivalve RNA-seq	Total Viral Reads	% of Viral Reads *	NCBI ID	*Blastp*		
	Total	nr	Nearly Complete									*E*-Value	Description	Identity %
*Atrina pectinata*	17	15	1	mixed	China	PA	Atrpec_virus1	\	2104	0.00202	MG210792	0	Wenzhou picorna-like virus 26	99.57
*Crassostrea gigas*	148	109	13	gills	Italy	PA	Cragig_virus3	mytgal	898	0.00135	MG210795	\	\	\
						PA	Bivalve hepelivirus G	mytgal	4058	0.00611	KX158876	0	Bivalve hepelivirus G	99.95
						PA	Bivalve RNA virus G5	\	1382	0.00173	KX158874	0	Bivalve RNA virus G5	100
						PA	Bivalve RNA virus G3	mytgal	1286	0.00161	KX158873	0	Bivalve RNA virus G3	100
						PA	Cragig_virus1	\	890	0.00134	MG210793	\	\	\
						PA	Cragig_virus2	mytgal	468	0.00058	MG210794	0	Wenzhou picorna-like virus 24	93.6
						PA	Bivalve RNA virus G1	mytgal	37902	0.04732	KX158871	0	Bivalve RNA virus G1	100
						PA	AY337486	mytgal	5156	0.00644	AY337486	0	Heterosigma akashiwo RNA virus	100
						RD	Cragig_virus6	\	30047	0.05554	MK561968	1^-108	Beihai picorna-like virus 21	66
						RD	Cragig_virus7	\	5493	0.01015	MK561969	3^-40	Wenzhou picorna-like virus 41	70
						RD	Cragig_virus8	\	955	0.00177	MK561970	0	Rhizosolenia setigera RNA virus	69
						RD	Cragig_virus9	\	9200	0.01701	MK561971	\	\	\
						RD	Cragig_virus10	\	568	0.00105	MK561972	\	\	\
*Elliptio complanata*	2	1	1	mixed	USA	PA	Elicom_virus1	\	2268	0.00552	MG210796	\	\	\
*Mizuhopecten yessoensis*	25	25	1	mixed	China	PA	Mizyes_virus1	\	1974	0.00522	MG210800	\	\	\
*Mytilus coruscus*			1	mixed	China	PA	Mytcor_virus1	\	4460	0.01028	MG210801	0	Pitaya virus X isolate P37	98.24
*Mytilus edulis*	37	33	1	mixed	France	PA	Mytedu_virus1	\	1936	0.00694	MG210802	0	Barns Ness breadcrumb sponge aquatic picorna-like virus 2	99
*Mytilus galloprovincialis*	115	52	3	gills	Italy	PA	Bivalve RNA virus G4	cragig, atrpec	4818	0.00713	KX158875	0	Bivalve RNA virus G4	99.77
						PA	Mytgal_virus1	cragig	\	\	MG210803	\	\	\
						PA	Mytgal_virus2	cragig	1432	0.00212	MG210804	0	Wenzhou picorna-like virus 51	78.7
*Ruditapes philippinarum*	121	49	5	gills	China	PA	Rudphi_virus1	\	9842	0.02869	MG210805	0	Wenzhou picorna-like virus 38	72.9
				gills	China	PA	Rudphi_virus2	\	1031	0.00301	MG210806	\	\	\
				gills	China	PA	Rudphi_virus3	\	181992	0.29884	MG210807	0	Wenzhou gastropodes virus 2	97.3
				gills	China	PA	Rudphi_virus4	ruddec, ostste, ostlur, cracor, cragig, mytedu	5388365	3.06157	MG210808	0	Wenzhou gastropodes virus 1	92
				larvae	USA	PA	Rudphi_virus5	\	19965	0.02936	MG210809	0	Marine RNA virus BC-4	70

Abbreviations: ruddec, *R. decussatus*; rudphi, *R. philippinarum*; ostste, *O. stentina*; ostlur, *O. lurida*; cragig, *C. gigas*; atrpec, *A. pectinata*; mytgal, *M. galloprovincialis*; mytedu, *M. edulis*; PA, polyadenylated RNA library; RD, ribo-depleted RNA library; * percentage of reads from the RNA-seq sample with the highest number of mapped reads, mapping to the given virus (counting only correctly paired reads).

## Data Availability

Short RNA-sequencing reads have been deposited in the NCBI SRA archive with accession ID SRR8587800, as part of the SRA project PRJNA484109.

## Supplementary Materials

The following are available online at https://www.mdpi.com/1999-4915/11/3/205/s1, Supplementary Table S1. Summary of the sequence data used for this work. 1. Genome data of bivalves. Species name, order, genome status and source are reported. 2. Transcriptome data. Class, Organism abbreviation, project ID (SRA archive), species name and order, sample origin, tissue, read layout, and number of predicted CDS sequences are reported. 3. *C. gigas* transcriptome data used for RNA-seq expression analysis. Project and SRR IDs, description and millions of reads are reported. 4. Viral genomes. NCBI ID, genome size, virus name, and taxonomical classification are reported. Supplementary Table S2. Annotation of the 413 viral sequences. The E-value, accession, description, and identity of the first BLAST match are reported, as well as the PFAM domain identified on these sequences (region, E-value, PFAM accession, and name). Supplementary Table S3. Virus distribution over 250 RNA-seq samples. The number of reads mapping to the 26 viral genomes are reported for 250 RNA-seq samples. The 82 samples, including more than 1000 viral reads are highlighted in red. Supplementary Table S4. COI mapping results. Mapped reads (reported as per millions) for 16 RNA-seq samples on COI entries (only the entries with at least 0.01 % of mapped reads are reported). Supplementary Table S5. Expression data of 10 RNAi-associated genes in oyster RNA-seq samples. Expression data are reported as TPM. Gene ID, description, and RNA-seq sample IDs, grouped per experimental treatment, are reported. (Panel A-C) Amount of OsHV-1 reads or RNA virus reads are depicted as blue or red dots, respectively, and referred to a secondary log_10_ bar scale (on the right of each graph). Expression patterns of RNAi-associated genes in oyster RNA-seq samples. Supplementary Figure S1. Genome construction of the 26 “nearly complete viral genomes” described in this work. ORFs are depicted as green arrows, and polyadenylation tails as black boxes. Supplementary Data S1. Phylogenetic tree of vRdRP regions in nexus format.
