# Supplementary figures and images for: A Needle in A Haystack: Tracing Bivalve-Associated Viruses in High-Throughput Transcriptomic Data
Source: Viruses. 2019 Mar 1;11(3):205. doi: 10.3390/v11030205 (PMC6466128; doi:10.3390/v11030205)

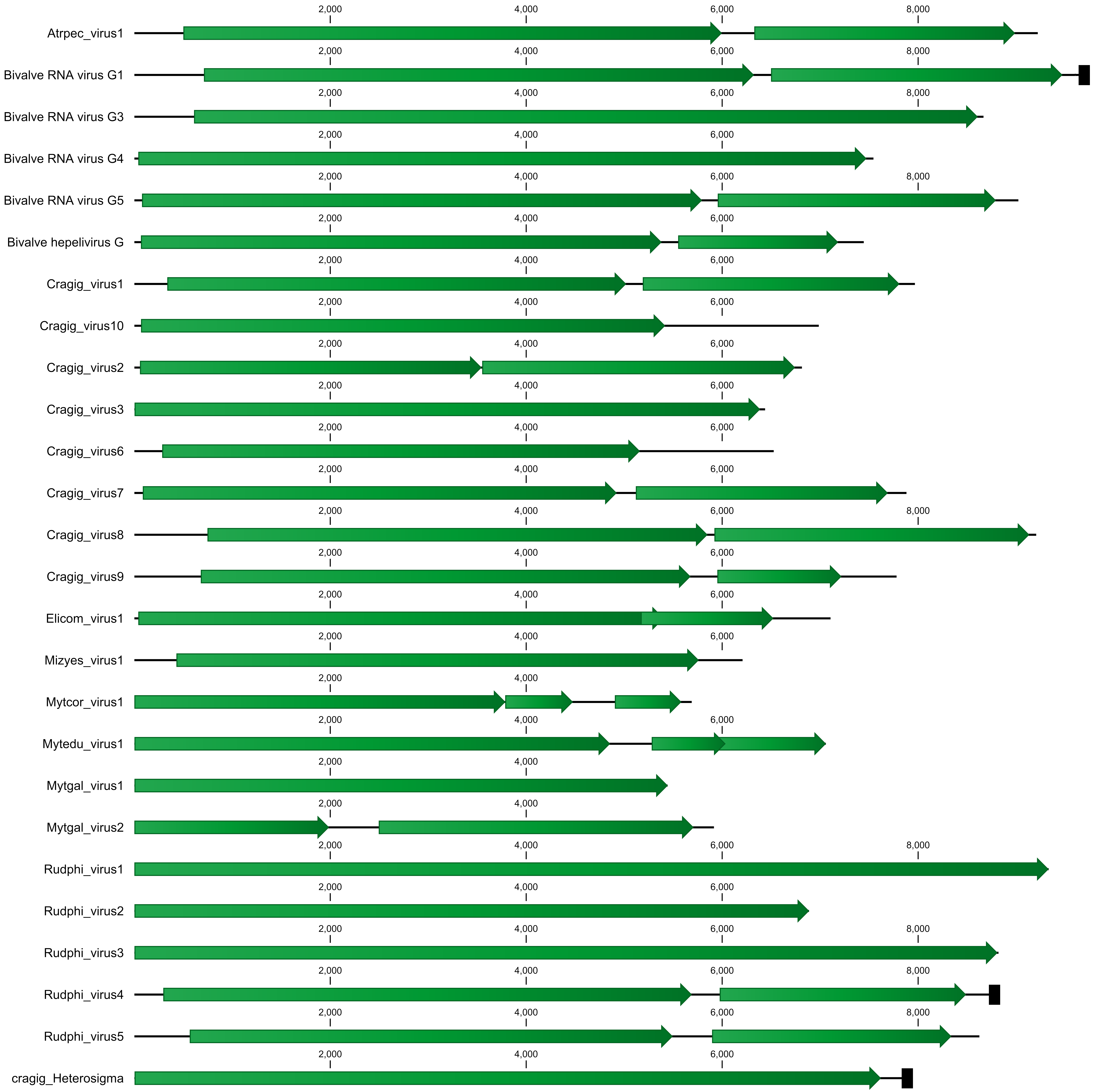

Supplement: Supplementary file 1 [file viruses-11-00205-s001.zip › S. Figure 1.tif]
